# Systematic review of the utility of the frailty index and frailty phenotype to predict all-cause mortality in older people

**DOI:** 10.1186/s13643-022-02052-w

**Published:** 2022-09-02

**Authors:** Dani J. Kim, M. Sofia Massa, Caroline M. Potter, Robert Clarke, Derrick A. Bennett

**Affiliations:** 1 CTSU, Nuffield Department of Population Health, University of Oxford, Big Data Institute, Old Road Campus, Oxford, OX3 7LF UK; 2 Health Services Research Unit (HSRU), Nuffield Department of Population Health, University of Oxford, Oxford, UK

**Keywords:** Frailty, Predictive ability, Discrimination, All-cause mortality, Frailty index, Frailty phenotype

## Abstract

**Background:**

Current guidelines for healthcare of community-dwelling older people advocate screening for frailty to predict adverse health outcomes, but there is no consensus on the optimum instrument to use in such settings. The objective of this systematic review of population studies was to compare the ability of the frailty index (FI) and frailty phenotype (FP) instruments to predict all-cause mortality in older people.

**Methods:**

Studies published before 27 July 2022 were identified using Ovid MEDLINE, Embase, Scopus, Web of Science and CINAHL databases. The eligibility criteria were population-based prospective studies of community-dwelling older adults (aged 65 years or older) and evaluation of both the FI and FP for prediction of all-cause mortality. The Scottish Intercollegiate Guidelines Network’s Methodology checklist was used to assess study quality. The areas under the receiver operator characteristic curves (AUC) were compared, and the proportions of included studies that achieved acceptable discriminatory power (AUC>0.7) were calculated for each frailty instrument. The results were stratified by the use of continuous or categorical formats of each instrument. The review was reported in accordance with the PRISMA and SWiM guidelines.

**Results:**

Among 8 studies (range: 909 to 7713 participants), both FI and FP had comparable predictive power for all-cause mortality. The AUC values ranged from 0.66 to 0.84 for FI continuous, 0.60 to 0.80 for FI categorical, 0.63 to 0.80 for FP continuous and 0.57 to 0.79 for FP categorical. The proportion of studies achieving acceptable discriminatory power were 75%, 50%, 63%, and 50%, respectively. The predictive ability of each frailty instrument was unaltered by the number of included items.

**Conclusions:**

Despite differences in their content, both the FI and FP instruments had modest but comparable ability to predict all-cause mortality. The use of continuous rather than categorical formats in either instrument enhanced their ability to predict all-cause mortality.

**Supplementary Information:**

The online version contains supplementary material available at 10.1186/s13643-022-02052-w.

## Background

Frailty is a state of vulnerability to external stressors in older people that reduces their resilience and ability to deal with stress [1–4]. Multiple instruments have been advocated to detect frailty in clinical practice, both in primary care [5] and hospital settings [6, 7], in order to identify individuals at high risk of suffering adverse health outcomes [3, 4, 8]. The two most widely used approaches to detect frailty are the frailty index (FI) [4] and the frailty phenotype (FP) [2] instruments, and each of these instruments have distinct, albeit complementary, features [9]. The FI defines frailty as a state of age-related accumulation of deficits and is measured as a ratio of deficits detected (usually 30 or more age-related health indicators that cover a range of domains) [10] to the total number of health indicators considered [11]. The FP, based on the phenotype of frailty model, characterises frailty as a syndrome involving five physical characteristics (weight loss, weakness, exhaustion, slowness and low activity) and is associated with reduced levels of energy and reserve [2]. In addition, each frailty instrument can vary depending on the type and format of the variables used for each instrument.

Despite their widespread use, the selection of FI over FP, or vice versa, by researchers and clinicians is often a pragmatic rather than being an evidence-based choice. Moreover, there is no consensus on the optimum model to detect frailty in population-based observational studies or in clinical practice. Overall, there is little available evidence directly comparing the discrimination, accuracy [12] or reliability [13, 14] of the most widely used frailty instruments for prediction of all-cause mortality.

Previous studies that compared the ability of different frailty instruments to predict all-cause mortality in older people reported that the FI was a slightly better predictor of all-cause mortality than the FP [15–18]. However, differences in the methodology used in the different studies limited direct comparisons of the diagnostic utility of each frailty instrument. Previous studies were also constrained by comparisons of studies conducted in diverse settings or involving populations with different absolute risks of all-cause mortality [15, 16]. The heterogeneity in the different approaches used to detect frailty and the statistical methods used to analyse discrimination precluded reliable comparisons [15, 17, 19]. Frailty instruments differ substantially in the number of items and domains included, but the findings from these different instruments are often used interchangeably or directly compared without appropriate recognition of the magnitude of differences between studies. Therefore, restricting the comparisons to fewer instruments and to comparable population settings may help to address the limitations and enable comparisons of the discriminative ability of different frailty instruments to predict all-cause mortality. The aims of the present report were to conduct a systematic review of prospective studies that investigated both FI and FP and to compare their ability to predict all-cause mortality in community-dwelling older people.

## Methods

The findings were reported according to the Preferred Reporting Items for Systematic Reviews and Meta-Analyses (PRISMA) [20]: Additional file 1: Table S1) and Synthesis Without Meta-analysis (SWiM) [21]. The Cochrane Library and PROSPERO international prospective register of systematic reviews were searched for similar reviews. A protocol was not registered for this review.

### Data sources

We searched the Ovid MEDLINE, Embase, Scopus, Web of Science and CINAHL databases for population studies of frailty in older people that were conducted between 1 January 2000 (shortly before the initial reports of each frailty instrument) and 22 January 2021. Further literature searches conducted on 21 September 2021 and 26 July 2022 did not identify any additional studies.

### Search strategy and selection criteria

The search strategy pre-specified the following components: (i) prospective cohort studies, (ii) evaluation of both frailty instruments and (iii) restrictions to studies reported in the English language (Additional file 1: Table 2). Full texts were retrieved if the study’s eligibility could not be determined by review of the abstracts. Studies were eligible for inclusion if they involved: (i) population-based prospective studies of community-dwelling older people (aged ≥65 years) excluding individuals recruited from long-term care facilities or hospital settings, (ii) compared instruments that defined frailty according to the Accumulation of Deficits (FI) and the Phenotype of Frailty (FP) models and (iii) used receiver operating characteristic (ROC) curves to compare frailty instruments for prediction of all-cause mortality. The study selection was carried out by a single reviewer (DJK), but the data extraction and quality assessment were conducted independently by two reviewers (DJK and MSM).

### Quality assessment

The Scottish Intercollegiate Guidelines Network’s (SIGN) Methodology checklist [22, 23] for prospective cohort studies was used to classify the methodological quality of the included studies [24]. The checklist included standardised statements to assess possible risks of bias in individual studies, including selection of participants, definition of exposure and outcomes, control of confounding and statistical analyses. All studies were rated using 14 categories of methodological quality (Additional file 1: Table S3), which were used to grade the overall confidence in the results of studies as either high-quality (++), acceptable (+) or low-quality (0) ratings.

### Data extraction

Two reviewers (DJK and MSM) independently extracted the data using a standardised data extraction form (Additional file 1: Table S4). The data were initially extracted on the first author, publication year, country and name of study, sample size, length of follow-up, participant characteristics (average age, % male), methodological quality and risk of bias, and methods used for prediction of all-cause mortality (e.g. AUC [95% CI]). The data extraction form was then updated to also include the number of deaths, level of adjustment for confounders and type of regression models used to estimate the AUC. When multiple adjustments for confounders were used, AUC estimates based on the most comprehensive adjustment were extracted. If results for multiple follow-up periods were reported, the data were extracted for the duration of follow-up that was most widely used in all included studies. Disagreements were resolved by consensus and, if still unresolved, were moderated by a third reviewer (RC). Finally, details of how each frailty instrument was estimated (e.g. the list of items included in the FI-based instruments and the criteria used to define each FP component) were recorded and supplemented by review of published cohort profiles (or contacting authors) for further information if needed.

The FI, estimated using a ratio (range 0–1), or the FP, using ordinal score (range 0–5), can also be assessed using a categorical format with binary (non-frail or frail) or 3 levels (non-frail, pre-frail and frail). For example, the values for FI ratio greater than 0.25 or an FP score greater or equal than 3 (out of 5 items) are typically defined as being frail [2, 4]. Such categorisations can lead to loss of information and reduce the power to detect associations between frailty measurements and adverse health outcomes [25], in addition to the reductions in their predictive ability. Therefore, to assess the predictive ability of the FI and FP for all-cause mortality, we recorded whether the instruments were used in a continuous or categorical format, and the data were extracted separately for each format.

### Data synthesis and analysis

The extracted data were compared in a descriptive manner. A formal meta-analysis was not considered appropriate because of the substantial methodological heterogeneity between the individual studies [26]. The Cochrane Handbook outlines methods to synthesise findings without conducting a meta-analysis [27]. In addition, the present review adhered to the reporting methodology outlined for data synthesis without meta-analysis (SWiM) guidelines [21]. The SWiM guideline is a 9-item reporting checklist that provides a standardised approach to reporting alternative synthesis methods.

For each instrument, the results of individual studies were classified by the instrument type, as either continuous or categorical format. The AUC was used as the standardised metric to compare the predictive ability of frailty instruments [26, 28]. In cases of incomplete data, the authors were contacted to supply the data or AUCs were approximated using sensitivity and specificity if provided. AUCs were displayed using a forest plot, and their range was reported by instrument model and type. We calculated the proportion of results that met the criteria of having acceptable discriminatory power (AUC≥0.7) and compared the summary statistics by instrument model and type. An AUC of ≥0.7 indicated that there was a 70% chance that the frailty instrument could rank a person who died with a higher frailty score than a person who survived. Although no restrictions were made on reporting of results, the quality of studies was determined using the SIGN checklist tool and displayed alongside the results.

Study results were displayed using a forest plot to allow the reader to visually inspect heterogeneity between results of individual studies. Further visual inspection of the AUCs was carried out by ordering or labelling the forest plot by study characteristics and using funnel plots. We examined whether AUCs between studies differed by study quality, number of deaths, level of adjustment for confounders, duration of follow-up and characteristics of the frailty instruments (for the FI model, the number of items, or for the FP model, domains included and threshold used to define frail). The domains considered for these analyses were adapted from a previous report [29] and included energy, physical activity, weight loss/BMI, strength, gait-related, mood, activities of daily living (ADL), self-rated health, hearing and vision, incontinence, medication, sleep, hospitalisation, comorbidities, symptoms, social support and falls.

## Results

### Study selection and characteristics

**Fig. 1:**
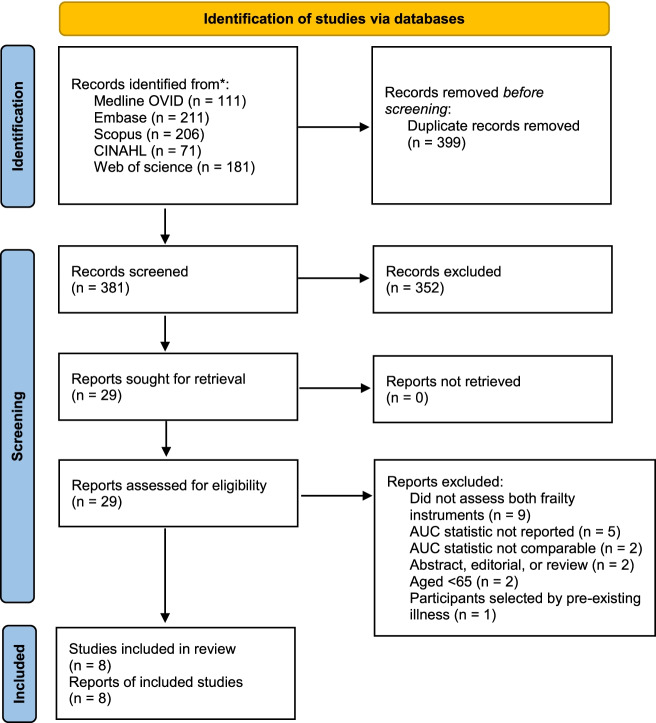
PRISMA 2020 flow diagram of included studies. *Search was carried out from 1 January 2000 to 22 January 2021. Update searches were carried out on 21 September 2021 and 26 July 2022, but did not identify any more eligible studies

The systematic review was documented using a PRSIMA 2020 flow diagram (Fig. 1). The initial search identified 780 reports, which included 399 duplicate studies. After review of the title and abstracts, we identified 29 reports for detailed assessment of eligibility for inclusion in the present review. Of the 10 community-based prospective cohort studies that were eligible for analysis, we were unable to assess the AUC from 2 studies where the non-frail participants were excluded from the analysis [30] or the pre-frail and frail categories were combined [31]. In total, 8 studies were included in the present review.

Selected characteristics of the 8 included studies [32–39] are presented in Table 1. The number of participants in the individual studies varied from 909 to 7713, and their mean age varied from 69.4 to 81.1 years. The duration of follow-up for all-cause mortality of the included AUC estimates varied from 2 to 7 years. Most studies involved participants living in Europe (*N*=3) [33, 34, 37] or Australia (*N*=2) [38, 39], and the remaining 3 studies involved participants living in the USA [32], China [35] or multiple diverse populations in Europe, North America and Australia [36].

**Table 1:** Characteristics of included studies by study size and details of the frailty index used

Author (year)	Country (name of study)^**a**^	Size (% male)	Age, years mean (SD)	Follow-up (years)	Study quality (SIGN^b^ checklist)	No. of items	Details of the frailty index used in individual studies					
							Domains included					
							Energy	Physical activity	Weight loss or BMI	Strength^c^	Gait ^**d**^	No. of other domains^**e**^
Chao (2018) [32]	USA (HRS)	FP: 1642 (n/s); FI: 7713 (n/s)	n/s (aged ≥65)^f^	7	+	24	✓					8
Romero-Ortuno and Soraghan (2014) [33]	Europe - various (SHARE)	7058 (43.3%)	M: 80.4 (4.6); F: 81.1 (4.9)	5	++	70	✓	✓		✓	✓	12
Ding (2017) [34]	England (ELSA)	4638 (44.6%)	74.0 (6.3)	2	0	30	✓			✓	✓	6
Woo (2012) [35]	China	4000 (50%)	n/s (aged ≥65)	4	+	47		✓	✓	✓	✓	8
Li (2015) [36]	Europe, North America and Australia (GLOW)	3985 (0%)	69.4 (8.9)	3	++	34	✓	✓	✓	✓	✓	7
Zucchelli (2019) [37]	Sweden (SNSACK)	3363 (35.1%)	74.7 (11.2)	3 to 5	+	45					✓	10
Widagdo (2015) [38]	Australia (ALSA)	2087 (n/s)	n/s (aged ≥65)	3	0	39				✓	✓	8
Thompson (2019) [39]	Australia (NWAHS)	909 (45%)	74.4 (6.2)	1 to 10	++	34	✓	✓	✓	✓	✓	8

^a^Study names are as follows: *SHARE*, The Survey of Health, Ageing and Retirement in Europe; *HRS*, The Health and Retirement Study; *ELSA*, The English Longitudinal Study of Ageing; *GLOW*, The Global Longitudinal Study of Osteoporosis; *SNSACK*, The Swedish National Study on Ageing and Care in Kungsholmen; *ALSA*, The Australian Longitudinal Study of Ageing; *NWAHS*, The North West Adelaide Health Study

^b^*SIGN*, Scottish Intercollegiate Guidelines Network. ^++^all or most of the criteria in the SIGN checklists have been fulfilled, ^+^some of the criteria have been fulfilled, ^0^few or no criteria fulfilled

^c^Items like grip strength and difficulty lifting weights over 10 lbs

^d^Items like gait speed, can walk 100m, difficulty with moving around, and usage of walking stick

^e^See Additional file 1: Table S6 for full list of domains (18 domains: energy, physical activity, weight loss/BMI, strength, gait, cognition, mood, activities of daily living, self-reported health, hearing and vision, incontinence, medication, sleep, hospitalisation, comorbidities, symptoms, social support and falls)

^f^HRS includes aged 50 or older participants at baseline but the author limits the analysis to those aged 65 and older

### Quality assessment

According to the SIGN checklist, 3 reports were rated as having a ‘high quality (++)’ [33, 36, 39], 3 had ‘acceptable quality (+)’ [32, 35, 37] and 2 had a ‘low-quality score (0)’ [34, 38]. The risk of bias chiefly reflected uncertainty about the response rates and loss to follow-up by levels of frailty (Additional file 1: Table S5).

### Comparative ability of FI and FP to predict all-cause mortality

Eight studies compared the predictive ability of FI and FP for all-cause mortality (the extracted data are presented in Additional file 1: Tables S6 and S7). Of these, 3 studies assessed the frailty instruments using a continuous format, 1 study using categorical format and 4 studies involved both continuous and categorical formats (Additional file 1: Table S8). Two studies reported AUCs separately by sex [33, 35], and one study was restricted to female-only participants [36].

**Fig. 2:**
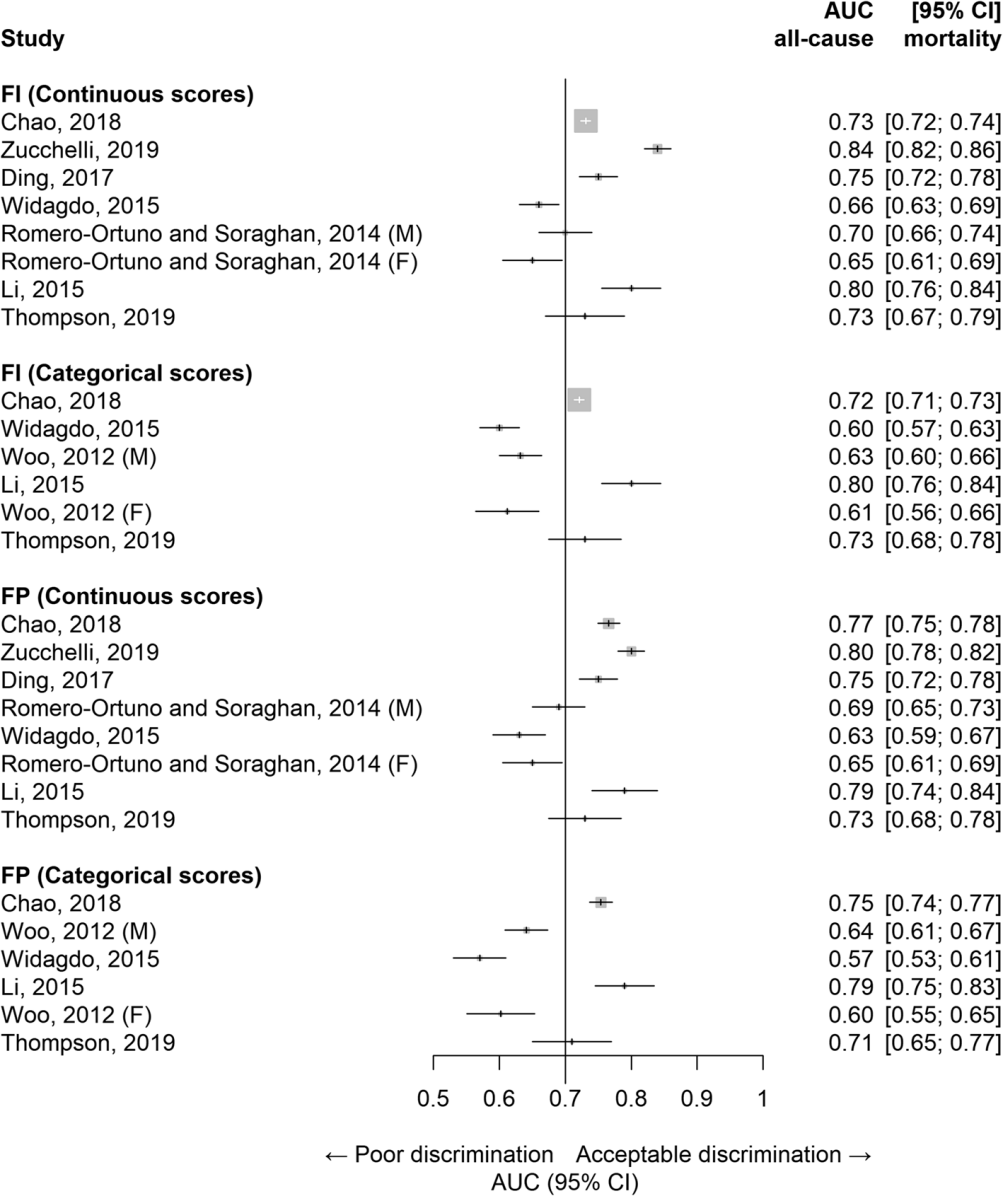
Discrimination assessed using area under the curve (AUC) estimates for prediction of all-cause mortality in included studies

The AUCs using both the FI and FP for prediction of all-cause mortality are shown in Fig. 2. The range of AUCs (and their respective 95% CIs) were 0.65 (95% CI 0.61–0.70) to 0.84 (0.82–0.86) for FI continuous, 0.60 (0.57–0.63) to 0.80 (0.75–0.84) for FI categorical, 0.63 (0.59–0.67) to 0.80 (0.78–0.82) for FP continuous and 0.57 (0.53–0.61) to 0.79 (0.75–0.83) for FP categorical, respectively. Likewise, the proportions of study results exceeding an AUC threshold ≥0.70 for acceptable discrimination were 75% (6/8), 50% (3/6), 63% (5/8) and 50% (3/6) for the FI continuous, FI categorical, FP continuous and FP categorical scores, respectively. The proportion of results that reached this threshold for acceptable discriminatory ability was higher for FI than for FP and for frailty instruments used in continuous rather than categorical forms. The distribution of AUC values was lower for those that used categorical rather than continuous formats of the frailty instruments.

### Assessment and exploration of heterogeneity

The duration of follow-up of the included studies varied from 2 to 7 years. The methods used to record deaths differed by study and included proxy-reported [32, 34, 36] or linkage to national death registers [37–39]. The definition of frailty instruments also varied among studies that reported using same frailty model.

No two FP-based instruments were identical, and all the FP instruments included in the review were modifications of the approach proposed by the original authors [2] (Additional file 1: Table S9). Many of the modifications involved minor differences in the survey used to define the FP components. For example, weight loss was defined using various thresholds (greater than 5% or 1, 3, 4.5 or 5 kg) of weight loss or BMI (<18.5 or 21kg/m^2^) or using self-reported questions (“Did you suffer from weight loss..?” or “What has your appetite been like?”). The chief modification involved defining FP as a factor score identified using confirmatory factor analysis [34]. Most of the FP-based instruments involved a combination of self-reported and objective measures as originally developed, but the instruments operationalised by Li et al. (2015) used self-reported measures for all five components (weight loss, weakness, exhaustion, slowness and low activity) [36].

The number of items (range 24–70) and domains included for instruments developed from the FI model also varied (Additional file 1: Tables S10 and S11). Most instruments were constructed using the systematic procedure developed by the original authors [10] and were multidimensional. All but 3 studies [32, 34] included at least 30–40 items as suggested in the systematic procedure, though no fixed number of items is established for the FI model. In the studies included in this review, the operationalisation of each FI instrument included activities of daily living (ADL) and comorbidity domains. In addition, the five FP domains were included in FI instrument to varying degrees (Table 1): the slow walking speed domain was included in most instruments, whereas weight loss was included in fewer instruments. Two studies defined FI that included all 5 FP domains [36, 39], but other studies included only one domain [32, 37]. Li and colleagues (2015) also defined continuous FI scores using quintiles rather than the number of items [36]. Furthermore, the thresholds used to detect frailty varied between studies (either 0.2, 0.25 or 0.35).

The statistical methods used to derive the AUC statistics also differed. Most studies used logistic regression [32–36, 38] or Cox regression [39], one study conducted a non-parametric ROC analysis [37] and one study did not provide details of the methods used [30]. The level of adjustment for confounders also varied between studies (Additional file 1: Tables S8 and S9).

The forest plot shows poor overlap in the 95% confidence intervals for AUC of the individual studies, indicating substantial statistical heterogeneity. To explore whether differences in discrimination were correlated with the number of outcomes included or study quality, we plotted the AUCs against the number of deaths and study quality (Additional file 1: Figure S1). The funnel plot shows that studies reporting AUC≥0.7 either had a larger number of outcomes (>500 deaths) or their quality score was high, except for one study [34], which used a modified frailty measure (based on factor scores), had a smaller number of events and a low study quality score. Although not pre-specified, a subgroup analysis excluding studies with low quality did not change the summarised range, but the proportions of study results exceeding an AUC threshold ≥0.70 were 83%, 60%, 66% and 60% for the FI continuous, FI categorical, FP continuous and FP categorical scores, respectively. Additional stratification by number of confounders adjusted for or by duration of follow-up did not influence AUCs for all-cause mortality (data not shown).

**Fig. 3:**
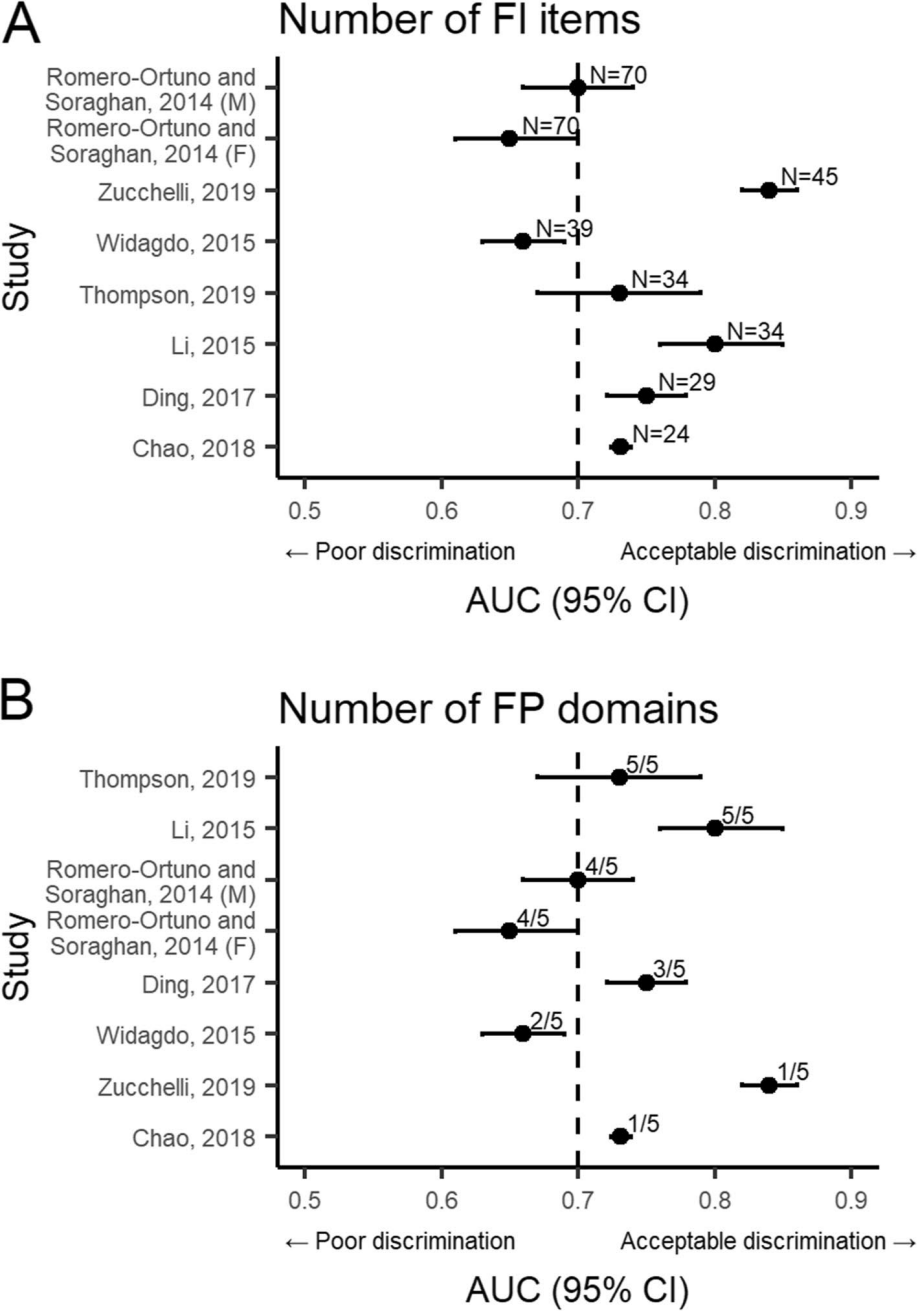
Discrimination of all-cause mortality assessed using area under the curve (AUC) of frailty index (FI) score by **A** FI items and **B** frailty phenotype domains included

Given the substantial differences in the FI-based instruments, we explored whether the number of items and domains included in the index were related to the discriminative ability of continuous FI scores (Fig. 3), but no evidence of such patterns were detected. The total number of domains or the cut-off thresholds used (for categorical FI) did not alter their predictive value for all-cause mortality (Additional file 1: Figures S2 and S3). Overall, both FP and FI had comparable, albeit only modest, ability to predict all-cause mortality.

## Discussion

Frailty is a well-established risk factor for adverse health outcomes, and assessments of frailty are widely used to guide multiple clinical decisions in older people in addition to prediction of all-cause mortality. However, the heterogeneity between results obtained using the available instruments to detect frailty has resulted in substantial uncertainty for both clinicians and researchers about the optimum instrument, or conceptual model, to use to assess frailty [9, 28, 40–42]. Previous systematic reviews had suggested that the FI instrument may be superior to FP for prediction of all-cause mortality [15–18]. Despite substantial differences in their content, the present systematic review demonstrated that both the FP and FI instruments had modest but comparable ability to predict all-cause mortality.

The novel aspect of the review was the inclusion of direct comparisons of the frailty models using results obtained from the same individuals in different studies (i.e., with comparable selection biases and absolute risks for all-cause mortality). This approach should enhance the reliability of the comparisons outlined in the present study [12, 15–18, 43].

The present review also explored the determinants of the predictive ability of frailty instruments. Continuous formats of the frailty instruments had slightly superior discrimination compared with their categorical formats (albeit these results were based on fewer studies). Alternatively, the number of items [10, 44] or the type of domains included in the FI-based instruments did not influence the discriminative ability of the instruments. The domains included in the FI-based instruments were wide-ranging, and the most commonly included were ADL and comorbidities. The FP domains were also included in the FI instruments to varying degrees, but it was difficult to ascertain which were the most informative domains. The reason that the FI was not superior to FP for prediction of mortality, despite including more items and domains (possibly being a more accurate reflection of the multidimensional frailty construct), may reflect the greater within-person variability of frailty measurements by FI that may have attenuated its association with mortality [45]. However, there is no consensus on the reliability of different frailty models for prediction of mortality. Instead, it is possible that the FI and FP are actually measuring different constructs [46]: an idea that is supported by the limited overlap between the two constructs within individual populations [47].

Both the FI and FP models are susceptible to misclassification bias, which may explain the modest predictive ability for either model [32]. The loss of information by arbitrary classification of continuous variables and inter-operator variability in variables such as grip strength may introduce misclassification bias and reduce the statistical power to detect associations with mortality [25]. If fewer frail cases are correctly identified, this misclassification may have underestimated the strength of associations [48]. Consistent with this, frailty indices involving fewer items [32] or individual domains [49] and self-reported frailty phenotype domains [50] have been shown to improve the prediction of all-cause mortality compared with the original versions in the same population. The present review, which compared predictive ability across populations, did not find such patterns, perhaps reflecting heterogeneity between results of different studies that may have obscured any true differences.

The chief strengths of the present review were the synthesised results based on reports involving a large number of participants and were mainly of high methodological quality. The methodological quality of reports was assessed using a standardised checklist and used to explore inconsistencies in the results. Data extraction and quality assessment were carried out by two independent reviewers and the search strategy should be reproducible. Nevertheless, the study had several limitations. First, the substantial methodological heterogeneity across studies may have obscured true differences and constrained the strength of the conclusions that can be inferred from the present study. Each instrument included several modifications and such differences limited the validity of the comparisons between studies. We have reported any discrepancies to illustrate the magnitude of heterogeneity to be considered when performing a systematic review of these frailty instruments. Second, the review was limited to studies that compared two frailty instruments in the same population, which allowed for a more direct comparison, but excluded studies using only one of the instruments. Moreover, the present review was also constrained by limiting the inclusion criteria to studies that reported their findings in the English language. Finally, the small number of studies included meant that while investigation of heterogeneity and grouping of results from individual studies was possible, synthesised findings should be interpreted with caution. For instance, fewer and different studies were included in the categorical than in continuous subgroups, which makes the comparison of proportion of studies exceeding the AUC threshold less robust.

Overall, there is still considerable uncertainty about the optimum approach to screen for frailty. However, the present study demonstrated that use of continuous rather than categorical frailty scores may enhance their ability to predict adverse outcomes. We identified a substantial heterogeneity in the application of frailty instruments in individual studies, which limited our comparative analyses. The variation between populations studied and their diverse healthcare settings constrain comparisons of the original frailty instruments. Future systematic reviews could instead compare the precise variations of a particular frailty instrument to identify the exact source of heterogeneity for each instrument. Such approaches could help identify the core domains of the FP or the number of deficits most suitable for the FI. In addition, establishing other important measurement properties of frailty instruments such as reliability, which may influence the magnitude of associations between frailty and adverse health outcomes [45, 51], could help to interpret differences in the performance of frailty measures.

## Conclusions

Despite the substantial differences in their content, the FI and FP had only modest but comparable ability to predict all-cause mortality in older people. We highlight an important and ongoing challenge in frailty research, which is the substantial heterogeneity in the definition of individual models. Further research is needed to determine the impact of such heterogeneity in the performance of the different frailty instruments by comparing the ability of individual frailty instruments in larger populations. The findings of these studies could inform the application of existing frailty instruments or possible modifications of existing instruments using electronic health records both in primary care and hospital settings to select the optimum instrument to detect frailty in older people.

## Supplementary Information

**Additional file 1: Table 1.** PRIMSA checklist. **Table 2.** Sample search strategy and results (MEDLINE OVID). **Table 3.** SIGN Methodology Checklist 3: Cohort studies. **Table 4.** Data extraction forms. **Table 5.** Rationale for SIGN checklist rating. **Table 6.** Characteristics of individual studies with data on FI. **Table 7.** Characteristics of individual studies with data on FP. **Table 8.** Rationale given by authors for continuous and categorical labels. **Table 9.** Details of the frailty phenotype (FP). **Table 10.** Details of the frailty index (FI). **Table 11.** Domains included in the FI. **Figure 1.** Plot of discriminative ability as assessed by Area Under the Curve (AUC) against number of events by methodological quality. **Figure 2.** Discriminative ability as assessed by Area Under the Curve (AUC) for Frailty Index (FI) continuous instruments arranged by total number of domains. **Figure 3.** Discriminative ability as assessed by Area Under the Curve (AUC) for Frailty Index (FI) categorical instruments.

## Data Availability

All data used in this review are available to bona fide researchers on request to the authors.

## Abbreviations

ADL: Activities of daily living
AUC: Area under the curve
BMI: Body mass index
FI: Frailty index
FP: Frailty phenotype
PRISMA: Preferred Reporting Items for Systematic Reviews and Meta-Analyses
PROSPERO: Prospective Register of Systematic Reviews
ROC: Receiver operating characteristic
SIGN: Scottish Intercollegiate Guidelines Network
SWiM: Synthesis Without Meta-analysis

## References

[1] Clegg A, Young J, Iliffe S, Rikkert MO, Rockwood K (2013). Frailty in elderly people. Lancet.

[2] Fried LP, Tangen CM, Walston J, Newman AB, Hirsch C, Gottdiener J (2001). Frailty in older adults evidence for a phenotype. J Gerontol Ser A.

[3] Bandeen-Roche K, Xue QL, Ferrucci L, Walston J, Guralnik JM, Chaves P (2006). Phenotype of frailty: characterization in the women’s health and aging studies. J Gerontol Ser A Biol Sci Med Sci.

[4] Mitnitski AB, Mogilner AJ, Rockwood K (2001). Accumulation of deficits as a proxy measure of aging. Scientific World J..

[5] Clegg A, Bates C, Young J, Ryan R, Nichols L, Ann Teale E (2016). Development and validation of an electronic frailty index using routine primary care electronic health record data. Age Ageing.

[6] Handforth C, Clegg A, Young C, Simpkins S, Seymour MT, Selby PJ (2015). The prevalence and outcomes of frailty in older cancer patients: a systematic review. Ann Oncol.

[7] Lin H-S, Watts JN, Peel NM, Hubbard RE (2016). Frailty and post-operative outcomes in older surgical patients: a systematic review. BMC Geriatr.

[8] Ofori-Asenso R, Chin KL, Sahle BW, Mazidi M, Zullo AR, Liew D (2020). Frailty confers high mortality risk across different populations: evidence from an overview of systematic reviews and meta-analyses. Geriatrics..

[9] Walston JD, Bandeen-Roche K (2015). Frailty: a tale of two concepts. BMC Med.

[10] Searle SD, Mitnitski A, Gahbauer EA, Gill TM, Rockwood K (2008). A standard procedure for creating a frailty index. BMC Geriatr.

[11] Rockwood K, Howlett SE (2018). Fifteen years of progress in understanding frailty and health in aging. BMC Med.

[12] Pijpers E, Ferreira I, Stehouwer CDA, Nieuwenhuijzen Kruseman AC (2012). The frailty dilemma. Review of the predictive accuracy of major frailty scores. Eur J Intern Med.

[13] Hoogendijk EO, Afilalo J, Ensrud KE, Kowal P, Onder G, Fried LP (2019). Frailty: implications for clinical practice and public health. Lancet.

[14] Nguyen QD, Moodie EM, Keezer MR, Wolfson C. Clinical correlates and implications of the reliability of the frailty index in the Canadian longitudinal study on aging. J Gerontol Ser A. 2021;(glab161) [cited 2021 Sep 21]. Available from. 10.1093/gerona/glab161. 10.1093/gerona/glab161 PMC8514068 34097017

[15] De Vries NM, Staal JB, Van Ravensberg CD, Hobbelen JSM, Olde Rikkert MGM, Nijhuis-Van Der Sanden MWG (2011). Outcome instruments to measure frailty: a systematic review. Ageing Res Rev.

[16] Bouillon K, Kivimaki M, Hamer M, Sabia S, Fransson EI, Singh-Manoux A (2013). Measures of frailty in population-based studies: an overview. BMC Geriatr.

[17] Dent E, Kowal P, Hoogendijk EO (2016). Frailty measurement in research and clinical practice: a review. Eur J Intern Med.

[18] Sutton JL, Gould RL, Daley S, Coulson MC, Ward EV, Butler AM, et al. Psychometric properties of multicomponent tools designed to assess frailty in older adults: a systematic review. BMC Geriatr. 2016;16(1). 10.1186/s12877-016-0225-2. 10.1186/s12877-016-0225-2 PMC4772336 26927924

[19] Pialoux T, Goyard J, Lesourd B (2012). Screening tools for frailty in primary health care: a systematic review. Geriatr Gerontol Int.

[20] Moher D, Liberati A, Tetzlaff J, Altman DG (2009). The PRISMA group. Preferred reporting items for systematic reviews and meta-analyses: the PRISMA statement. PLoS Med.

[21] Campbell M, McKenzie JE, Sowden A, Katikireddi SV, Brennan SE, Ellis S (2020). Synthesis without meta-analysis (SWiM) in systematic reviews: reporting guideline. BMJ..

[22] Petrie JC, Grimshaw JM, Bryson A (1995). The Scottish intercollegiate guidelines network initiative: getting validated guidelines into local practice. Health Bull (Edinb).

[23] Lowe G, Twaddle S (2005). The Scottish intercollegiate guidelines network (SIGN): an update. Scott Med J.

[24] Ma L-L, Wang Y-Y, Yang Z-H, Huang D, Weng H, Zeng X-T (2020). Methodological quality (risk of bias) assessment tools for primary and secondary medical studies: what are they and which is better?. Mil Med Res.

[25] Altman DG, Royston P (2006). The cost of dichotomising continuous variables. BMJ..

[26] Prinsen CAC, Mokkink LB, Bouter LM, Alonso J, Patrick DL, de Vet HCW (2018). COSMIN guideline for systematic reviews of patient-reported outcome measures. Qual Life Res Int J Qual Life Asp Treat Care Rehab.

[27] Higgins JPT, Chandler J, Cumpston M, Li T, Page M, Welch V (2021). Cochrane handbook for systematic reviews of interventions version 6.2.

[28] Rockwood K (2005). What would make a definition of frailty successful?. Age Ageing.

[29] Theou O, Brothers TD, Peña FG, Mitnitski A, Rockwood K (2014). Identifying common characteristics of frailty across seven scales. J Am Geriatr Soc.

[30] Op LPM, Beurskens AJHM, de Vet HCW, van Kuijk SMJ, Hajema K, Kempen GIJM (2019). The ability of four frailty screening instruments to predict mortality, hospitalization and dependency in (instrumental) activities of daily living. Eur J Ageing.

[31] Gonzalez-Colaco HM, Meillon C, Bergua V, Tabue TM, Dartigues J-F, Avila-Funes JA (2017). Comparing the predictive value of three definitions of frailty: results from the three-city study. Arch Gerontol Geriatr.

[32] Chao Y-S, Wu H-C, Wu C-J, Chen W-C (2018). Index or illusion: the case of frailty indices in the health and retirement study. Rogan S, editor. PLoS One.

[33] Romero-Ortuno R, Soraghan C (2014). A frailty instrument for primary care for those aged 75 years or more: findings from the survey of health, ageing and retirement in Europe, a longitudinal population-based cohort study (SHARE-FI75+). BMJ Open.

[34] Ding YY (2017). Predictive validity of two physical frailty phenotype specifications developed for investigation of frailty pathways in older people. Gerontology..

[35] Woo J, Leung J, Morley JE (2012). Comparison of frailty indicators based on clinical phenotype and the multiple deficit approach in predicting mortality and physical limitation. J Am Geriatr Soc.

[36] Li G, Thabane L, Ioannidis G, Kennedy C, Papaioannou A, Adachi JD. Comparison between frailty index of deficit accumulation and phenotypic model to predict risk of falls: data from the global longitudinal study of osteoporosis in women (GLOW) Hamilton cohort. PLoS One. 2015;10(3) [cited 2019 Nov 19]. Available from: https://www.ncbi.nlm.nih.gov/pmc/articles/PMC4357575/. 10.1371/journal.pone.0120144 PMC4357575 25764521

[37] Zucchelli A, Vetrano DL, Grande G, Calderon-Larranaga A, Fratiglioni L, Marengoni A (2019). Comparing the prognostic value of geriatric health indicators: a population-based study. BMC Med.

[38] Widagdo IS, Pratt N, Russell M, Roughead EE (2016). Construct validity of four frailty measures in an older Australian population: a rasch analysis. J Frailty Aging.

[39] Thompson MQ, Theou O, Tucker GR, Adams RJ, Visvanathan R. Recurrent measurement of frailty is important for mortality prediction: findings from the north West Adelaide health study. J Am Geriatr Soc. 2019. 10.1111/jgs.16066. 10.1111/jgs.16066 31317527

[40] Kusumastuti S, Gerds TA, Lund R, Mortensen EL, Westendorp RGJ (2017). Discrimination ability of comorbidity, frailty, and subjective health to predict mortality in community-dwelling older people: population based prospective cohort study. Eur J Intern Med.

[41] Gonzalez-Colaço Harmand M, Meillon C, Bergua V, Tabue Teguo M, Dartigues J-F, Avila-Funes JA (2017). Comparing the predictive value of three definitions of frailty: results from the three-city study. Arch Gerontol Geriatr.

[42] Xue Q-L, Varadhan R (2014). What is missing in the validation of frailty instruments?. J Am Med Dir Assoc.

[43] Sternberg SA, Schwartz AW, Karunananthan S, Bergman H, Clarfield AM (2011). The identification of frailty: a systematic literature review. J Am Geriatr Soc.

[44] Kojima G, Iliffe S, Walters K (2018). Frailty index as a predictor of mortality: a systematic review and meta-analysis. Age Ageing.

[45] Knuiman MW, Divitini ML, Buzas JS, Fitzgerald PEB (1998). Adjustment for regression dilution in epidemiological regression analyses. Ann Epidemiol.

[46] Cesari M, Gambassi G, van Kan GA, Vellas B (2014). The frailty phenotype and the frailty index: different instruments for different purposes. Age Ageing.

[47] Aguayo GA, Donneau A-F, Vaillant MT, Schritz A, Franco OH, Stranges S (2017). Agreement between 35 published frailty scores in the general population. Am J Epidemiol.

[48] Tripepi G, Jager KJ, Dekker FW, Zoccali C (2010). Selection bias and information bias in clinical research. Nephron Clin Pract.

[49] Chao Y-S, Wu C-J, Wu H-C, Hsu H-T, Tsao L-C, Cheng Y-P (2020). Composite diagnostic criteria are problematic for linking potentially distinct populations: the case of frailty. Sci Rep.

[50] Papachristou E, Wannamethee SG, Lennon LT, Papacosta O, Whincup PH, Iliffe S (2017). Ability of self-reported frailty components to predict incident disability, falls, and all-cause mortality: results from a population-based study of older British men. J Am Med Dir Assoc.

[51] MacMahon S, Peto R, Cutler J, Collins R, Sorlie P, Neaton J (1990). Blood pressure, stroke, and coronary heart disease: part 1, prolonged differences in blood pressure: prospective observational studies corrected for the regression dilution bias. Lancet.

