# Supplementary material for: Systematic review of the utility of the frailty index and frailty phenotype to predict all-cause mortality in older people
Source: Syst Rev. 2022 Sep 2;11:187. doi: 10.1186/s13643-022-02052-w (PMC9438224; doi:10.1186/s13643-022-02052-w)
Supplement: Supplementary file 1 — Additional file 1: Table 1. PRIMSA checklist. Table 2. Sample search strategy and results (MEDLINE OVID). Table 3. SIGN Methodology Checklist 3: Cohort studies. Table 4. Data extraction forms. Table 5. Rationale for SIGN checklist rating. Table 6. Characteristics of individual studies with data on FI. Table 7. Characteristics of individual studies with data on FP. Table 8. Rationale given by authors for continuous and categorical labels. Table 9. Details of the frailty phenotype (FP). Table 10. Details of the frailty index (FI). Table 11. Domains included in the FI. Figure 1. Plot of discriminative ability as assessed by Area Under the Curve (AUC) against number of events by methodological quality. Figure 2. Discriminative ability as assessed by Area Under the Curve (AUC) for Frailty Index (FI) continuous instruments arranged by total number of domains. Figure 3. Discriminative ability as assessed by Area Under the Curve (AUC) for Frailty Index (FI) categorical instruments. [file 13643_2022_2052_MOESM1_ESM.docx]

**Supplementary material for**

**Systematic review of the utility of the Frailty Index and Frailty Phenotype to predict all-cause mortality in older people**

Dani J. Kim, M. Sofia Massa, Caroline M. Potter, Robert Clarke, and Derrick A. Bennett

Table of Contents

[Appendix Table 1. PRIMSA checklist 2](#_Toc83111928)

[Appendix Table 2. Sample search strategy and results (Medline OVID) 5](#_Toc83111929)

[Appendix Table 3. SIGN Methodology Checklist 3: Cohort studies 7](#_Toc83111930)

[Appendix Table 4. Data extraction forms 9](#_Toc83111931)

[Appendix Table 5. Rationale for SIGN checklist rating 10](#_Toc83111932)

[Appendix Table 6. Characteristics of individual studies with data on FI 12](#_Toc83111933)

[Appendix Table 7. Characteristics of individual studies with data on FP 13](#_Toc83111934)

[Appendix Table 8. Rationale given by authors for continuous and categorical labels 14](#_Toc83111935)

[Appendix Table 9. Details of the frailty phenotype (FP) 15](#_Toc83111936)

[Appendix Table 10. Details of the frailty index (FI) 17](#_Toc83111937)

[Appendix Table 11. Domains included in the FI 20](#_Toc83111938)

[Appendix Figure 1. Plot of discriminative ability as assessed by Area Under the Curve (AUC) against number of events by methodological quality 21](#_Toc83111939)

[Appendix Figure 2. Discriminative ability as assessed by Area Under the Curve (AUC) for Frailty Index (FI) continuous instruments arranged by total number of domains 21](#_Toc83111940)

[Appendix Figure 3. Discriminative ability as assessed by Area Under the Curve (AUC) for Frailty Index (FI) categorical instruments 22](#_Toc83111941)

## Appendix Table 1. PRIMSA checklist

| **Section/topic** | **#** | **Checklist item** | **Reported on page #** |
| --- | --- | --- | --- |
| **TITLE** | | |  |
| Title | 1 | Identify the report as a systematic review, meta-analysis, or both. | 1 |
| **ABSTRACT** | | |  |
| Structured summary | 2 | Provide a structured summary including, as applicable: background; objectives; data sources; study eligibility criteria, participants, and interventions; study appraisal and synthesis methods; results; limitations; conclusions and implications of key findings; systematic review registration number. | 2 |
| **INTRODUCTION** | | |  |
| Rationale | 3 | Describe the rationale for the review in the context of what is already known. | 4,5 |
| Objectives | 4 | Provide an explicit statement of questions being addressed with reference to participants, interventions, comparisons, outcomes, and study design (PICOS). | 5,6 |
| **METHODS** | | |  |
| Protocol and registration | 5 | Indicate if a review protocol exists, if and where it can be accessed (e.g., Web address), and, if available, provide registration information including registration number. | Protocol was not published |
| Eligibility criteria | 6 | Specify study characteristics (e.g., PICOS, length of follow-up) and report characteristics (e.g., years considered, language, publication status) used as criteria for eligibility, giving rationale. | 6 |
| Information sources | 7 | Describe all information sources (e.g., databases with dates of coverage, contact with study authors to identify additional studies) in the search and date last searched. | 5,6 |
| Search | 8 | Present full electronic search strategy for at least one database, including any limits used, such that it could be repeated. | Appendix Table 2 |
| Study selection | 9 | State the process for selecting studies (i.e., screening, eligibility, included in systematic review, and, if applicable, included in the meta-analysis). | 6 |
| Data collection process | 10 | Describe method of data extraction from reports (e.g., piloted forms, independently, in duplicate) and any processes for obtaining and confirming data from investigators. | 7, Appendix Table 3, 4 |
| Data items | 11 | List and define all variables for which data were sought (e.g., PICOS, funding sources) and any assumptions and simplifications made. | 5 |
| Risk of bias in individual studies | 12 | Describe methods used for assessing risk of bias of individual studies (including specification of whether this was done at the study or outcome level), and how this information is to be used in any data synthesis. | 6, 8 |
| Summary measures | 13 | State the principal summary measures (e.g., risk ratio, difference in means). | 8 |
| Synthesis of results | 14 | Describe the methods of handling data and combining results of studies, if done, including measures of consistency (e.g., I^2^) for each meta-analysis. | 8 |
| Risk of bias across studies | 15 | Specify any assessment of risk of bias that may affect the cumulative evidence (e.g., publication bias, selective reporting within studies). | 8 |
| Additional analyses | 16 | Describe methods of additional analyses (e.g., sensitivity or subgroup analyses, meta-regression), if done, indicating which were pre-specified. | 8 |
| **RESULTS** | | |  |
| Study selection | 17 | Give numbers of studies screened, assessed for eligibility, and included in the review, with reasons for exclusions at each stage, ideally with a flow diagram. | 9, 24 |
| Study characteristics | 18 | For each study, present characteristics for which data were extracted (e.g., study size, PICOS, follow-up period) and provide the citations. | 9, 22 |
| Risk of bias within studies | 19 | Present data on risk of bias of each study and, if available, any outcome level assessment (see item 12). | 22 |
| Results of individual studies | 20 | For all outcomes considered (benefits or harms), present, for each study: (a) simple summary data for each intervention group (b) effect estimates and confidence intervals, ideally with a forest plot. | 25 |
| Synthesis of results | 21 | Present the main results of the review. If meta-analyses are done, include for each, confidence intervals and measures of consistency. | 9, 10 |
| Risk of bias across studies | 22 | Present results of any assessment of risk of bias across studies (see Item 15). | 11-13 |
| Additional analysis | 23 | Give results of additional analyses, if done (e.g., sensitivity or subgroup analyses, meta-regression [see Item 16]). | 12, 13, 26 |
| **DISCUSSION** | | |  |
| Summary of evidence | 24 | Summarize the main findings including the strength of evidence for each main outcome; consider their relevance to key groups (e.g., healthcare providers, users, and policy makers). | 13-15 |
| Limitations | 25 | Discuss limitations at study and outcome level (e.g., risk of bias), and at review-level (e.g., incomplete retrieval of identified research, reporting bias). | 15, 16 |
| Conclusions | 26 | Provide a general interpretation of the results in the context of other evidence, and implications for future research. | 15 |
| **FUNDING** | | |  |
| Funding | 27 | Describe sources of funding for the systematic review and other support (e.g., supply of data); role of funders for the systematic review. | 17 |

*From:*  Moher D, Liberati A, Tetzlaff J, Altman DG, The PRISMA Group (2009). Preferred Reporting Items for Systematic Reviews and Meta-Analyses: The PRISMA Statement. PLoS Med 6(7): e1000097. doi:10.1371/journal.pmed1000097

## Appendix Table 2. Sample search strategy and results (Medline OVID)

| **Searches** |  | **Results** |
| --- | --- | --- |
| 1 | epidemiologic studies/ | 8530 |
| 2 | follow-up studies/ or longitudinal studies/ or prospective studies/ or feasibility studies/ or multicenter studies as topic/ or twin studies as topic/ or epidemiological monitoring.mp. [mp=title, abstract, original title, name of substance word, subject heading word, floating sub-heading word, keyword heading word, organism supplementary concept word, protocol supplementary concept word, rare disease supplementary concept word, unique identifier, synonyms] | 1319134 |
| 3 | (cohort adj (study or studies or analys*)).ti,ab. | 233200 |
| 4 | ((follow up or observational or uncontrolled or non randomi#ed or nonrandomi#ed or epidemiologic*) adj (study or studies)).ti,ab. | 259582 |
| 5 | ((longitudinal or prospective) and (study or studies or review or analys* or cohort*)).ti,ab. | 739201 |
| 6 | or/1-5 | 1938363 |
| 7 | exp case control studies/ or exp control groups/ | 1137451 |
| 8 | cross-sectional studies/ or prevalence/ or ((cross-sectional or prevalence or transversal) adj3 (study or studies)).ti,ab. | 687687 |
| 9 | ((case* and control*) or (case* and comparison*) or control group*).ti,ab. | 1045791 |
| 10 | or/7-9 | 2574173 |
| 11 | 6 not 10 | 1403654 |
| 12 | ("Rockwood K" or "Mitnitski A").au. or (Rockwood or Mitnitski or "frailty index" or FI or "accumulated deficit*" or "cumulative deficit*" or "deficit accumulation").ti,ab. | 12181 |
| 13 | (frailty or "frail elderly").ti,ab. or Frailty/ or Frail Elderly/ | 23836 |
| 14 | 11 and 12 and 13 | 525 |
| 15 | (frailty or non-frail* or nonfrail or prefrail* or pre-frail* or frail*).ti,ab. or Frail Elderly/ | 28093 |
| 16 | (Fried or CHS or phenotyp* or syndrome* or "cardiovascular health study").ti,ab. or Phenotype/ or "Fried L".au. | 1646774 |
| 17 | (physical* adj3 frail*).ti,ab. | 1552 |
| 18 | (frail* adj3 criteri*).ti,ab. | 616 |
| 19 | (frail* adj3 defin*).ti,ab. | 1190 |
| 20 | or/16-19 | 1648520 |
| 21 | 11 and 15 and 20 | 1296 |
| 22 | 14 and 21 | 202 |
| 23 | exp mortality/ or exp fatal outcome/ or exp survival/ or exp survival analysis/ or exp prognosis/ or exp cause of death/ or (mortality or death* or died or dead or survival or prognosis or fatal*).ti,ab. | 3867502 |
| 24 | 22 and 23 | 118 |
| 25 | limit 24 to (english language and yr="2000 -Current") | 118 |
| 26 | (case reports or comment or editorial or letter or "review").pt. | 6452479 |
| 27 | 25 not 26 | 111 |

Search strategy adapted from the following sources:

1. Faller JW, Pereira DDN, De Souza S et al. Instruments for the detection of frailty syndrome in older adults: A systematic review. PLOS ONE 2019;14:e0216166.
2. Kojima G, Iliffe S, Walters K. Frailty index as a predictor of mortality: a systematic review and meta-analysis. *Age Ageing* 2018;**47**:193–200.
3. Theou O, Cann L, Blodgett J *et al.* Modifications to the frailty phenotype criteria: Systematic review of the current literature and investigation of 262 frailty phenotypes in the Survey of Health, Ageing, and Retirement in Europe. *Ageing Research Reviews* 2015;**21**:78–94.
4. National Guideline Centre (UK). *Literature Search Strategies*. National Institute for Health and Care Excellence (UK), 2016.

Search date: 22 January 2021

## Appendix Table 3. SIGN Methodology Checklist 3: Cohort studies

| **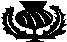 S I G N** | | | Methodology Checklist 3: Cohort studies | | | | |
| --- | --- | --- | --- | --- | --- | --- | --- |
| Study identification (*Include author, title, year of publication, journal title, pages* | | | | | | | |
| Guideline topic: | | | | Key Question No: | | Reviewer: | |
| **Before** completing this checklist, consider:  Is the paper really a cohort study? If in doubt, check the study design algorithm available from SIGN and make sure you have the correct checklist.  Is the paper relevant to key question? Analyse using PICO (Patient or Population Intervention Comparison Outcome). IF NO REJECT (give reason below). IF YES complete the checklist.. | | | | | | | |
| Reason for rejection: 1. Paper not relevant to key question □ 2. Other reason □ (please specify):  **Please note that a retrospective study (ie a database or chart study) cannot be rated higher than +.** | | | | | | | |
| Section 1: Internal validity | | | | | | | |
| ***In a well conducted cohort study:*** | | | | | **Does this study do it?** | | |
| 1.1 | | The study addresses an appropriate and clearly focused question. | | | Yes □  Can’t say □ | | No □ |
| Selection of subjects | | | | | | | |
| 1.2 | | The two groups being studied are selected from source populations that are comparable in all respects other than the factor under investigation. | | | Yes □  Can’t say □ | | No □  Does not apply □ |
| 1.3 | | The study indicates how many of the people asked to take part did so, in each of the groups being studied. | | | Yes □ | | No □  Does not apply □ |
| 1.4 | | The likelihood that some eligible subjects might have the outcome at the time of enrolment is assessed and taken into account in the analysis. | | | Yes □  Can’t say □ | | No □  Does not apply □ |
| 1.5 | | What percentage of individuals or clusters recruited into each arm of the study dropped out before the study was completed. | | |  | | |
| 1.6 | | Comparison is made between full participants and those lost to follow up, by exposure status. | | | Yes □  Can’t say □ | | No □  Does not apply □ |
| ASSESSMENT | | | | | | | |
| 1.7 | The outcomes are clearly defined. | | | | Yes □  Can’t say □ | | No □ |
| 1.8 | The assessment of outcome is made blind to exposure status. If the study is retrospective this may not be applicable. | | | | Yes □  Can’t say □ | | No □  Does not apply □ |
| 1.9 | Where blinding was not possible, there is some recognition that knowledge of exposure status could have influenced the assessment of outcome. | | | | Yes □  Can’t say □ | | No □  □ |
| 1.10 | The method of assessment of exposure is reliable. | | | | Yes □  Can’t say □ | | No □ |
| 1.11 | Evidence from other sources is used to demonstrate that the method of outcome assessment is valid and reliable. | | | | Yes □  Can’t say □ | | No □  Does not apply□ |
| 1.12 | Exposure level or prognostic factor is assessed more than once. | | | | Yes □  Can’t say □ | | No □  Does not apply □ |
| CONFOUNDING | | | | | | | |
| 1.13 | The main potential confounders are identified and taken into account in the design and analysis. | | | | Yes □  Can’t say □ | | No □ |
| STATISTICAL ANALYSIS | | | | | | | |
| 1.14 | Have confidence intervals been provided? | | | | Yes □ | | No □ |
| **Section 2: OVERALL ASSESSMENT OF THE STUDY** | | | | | | | |
| 2.1 | How well was the study done to minimise the risk of bias or confounding? | | | | High quality (++) □  Acceptable (+) □  Unacceptable – reject 0 | | |
| 2.2 | Taking into account clinical considerations, your evaluation of the methodology used, and the statistical power of the study, do you think there is clear evidence of an association between exposure and outcome? | | | | Yes □  Can’t say □ | | No □ |
| 2.3 | Are the results of this study directly applicable to the patient group targeted in this guideline? | | | | Yes □ | | No □ |
| 2.4 | **Notes.** Summarise the authors conclusions. Add any comments on your own assessment of the study, and the extent to which it answers your question and mention any areas of uncertainty raised above. | | | | | | |

From: https://www.sign.ac.uk/what-we-do/methodology/checklists/

## Appendix Table 4. Data extraction forms

##

| **Author, (year)** | **Country (Name of Study)** | **Size (male %)** | **Age, mean (SD)** | **Follow-up (y)** |
| --- | --- | --- | --- | --- |
|  |  |  |  |  |
|  |  |  |  |  |

| **Author, year** | **Size** | **Statistic reported** | **Results (estimate and confidence interval)** | **Adjustments in model** | **Result rating** | **Study quality (SIGN checklist)** | **Comments (if any, e.g. why results cannot be rated)** |
| --- | --- | --- | --- | --- | --- | --- | --- |
| **FI continuous** | | | | | | | |
|  |  |  |  |  |  |  |  |
|  | \|  \| \| --- \| |  |  |  |  |  |  |
| **FI categorical** | | | | | | | |
|  |  |  |  |  |  |  |  |
|  |  |  |  |  |  |  |  |
| **FP continuous** | | | | | | | |
|  |  |  |  |  |  |  |  |
|  |  |  |  |  |  |  |  |
| **FP categorical** | | | | | | | |
|  |  |  |  |  |  |  |  |
|  |  |  |  |  |  |  |  |

## Appendix Table 5. Rationale for SIGN checklist rating

Refer to Appendix Table 3 (page 7) for the SIGN checklist statement.

|  | **Chao (2018)** | **Romero-Ortuno and Soraghan (2014)** | **Ding (2017)** | **Woo (2012)** | **Li (2015)** | **Zucchelli (2019)** | **Op Het Veld (2019)** | **Widagdo (2015)** | **Gonzalez-Harmand (2017)** | **Thompson (2019)** |
| --- | --- | --- | --- | --- | --- | --- | --- | --- | --- | --- |
| **SECTION 1: INTERNAL VALIDITY** | | | | | | | | | | |
| **1.1** | Yes | Yes | Yes | Yes | Yes | Yes | Yes | Yes | Yes | Yes |
| **SELECTION OF SUBJECTS** | | | | | | | | | | |
| **1.2** | All ‘Can’t say’ – different frailty groups may not be comparable in all other respects. | | | | | | | | | |
| **1.3** | No | Yes | No | No | No | No | No | No | No | No |
| **1.4** | All ‘No’ – there is no likelihood that eligible subjects might have the outcome (mortality) at enrolment. | | | | | | | | | |
| **1.5** | No percentage | 23-34.3% | No percentage | No percentage | No percentage | No percentage | No percentage | No percentage | No percentage | No percentage |
| **1.6** | No | No | No | No | No | No | No | No | No | Yes |
| **ASSESSMENT** | | | | | | | | | | |
| **1.7** | Yes | Yes | Yes | Yes | Yes | Yes | Yes | Yes | Yes | Yes |
| **1.8** | Does not apply to statement ‘The assessment of outcome is made blind to exposure status’ because outcome (mortality) is completely objective. | | | | | | | | | |
| **1.9** | As above | | | | | | | | | |
| **1.10** | All ‘Can’t say’ because the reliability of exposure (frailty measurements) is uncertain | | | | | | | | | |
| **1.11** | Yes | Yes | Yes | Yes | Yes | Yes | Yes | Yes | Yes | Yes |
| **1.12** | No | No | No | No | No | No | No | No | No | Yes |
| **CONFOUNDING** | | | | | | | | | | |
| **1.13** | Yes | Yes | No | No | Yes | No | No | No | Yes | Yes |
| **STATISTICAL ANALYSIS** | | | | | | | | | | |
| **1.14** | Yes | Yes | No | Yes | Yes | Yes | No | No | Yes | Yes |
| **SECTION 2: OVERALL ASSESSMENT OF THE STUDY** | | | | | | | | | | |
| Notes | Overall response rate by wave reported elsewhere / Lost to f-u mitigated by active f-u / Missing values were imputed | Response rates reported elsewhere / Flow chart of study participants reported by frailty category / Compares baseline characteristic of participants with and without outcome. | Response rate by study wave reported elsewhere / Response rate is high / Missing values were imputed | Compared patient characteristics of those f-u and lost to f-u | Reports baseline characteristics by frailty group / Overall response rate at reported / Outcome self-reported but adjudication attempted / Missing values were imputed | Baseline response rate & attrition rate by age group reported elsewhere | Baseline response rate reported elsewhere / Missing values were imputed | Baseline response rate reported elsewhere / Sensitivity analysis carried out using participants with complete data | Death data systematically collected with a standardised interview | Compares frailty status at baseline and follow-up status by frailty categories. |
| # yes | 4 | 7 | 3 | 4 | 6 | 5 | 2 | 3 | 5 | 7 |
| 2.1 | + | ++ | 0 | + | ++ | + | 0 | 0 | + | ++ |

Rating given ++ if mostly ‘yes’ (6-7); + if some ‘yes’ (4-5); and 0 if few ‘yes’ (2-3).

## Appendix Table 6. Characteristics of individual studies with data on FI

| **Author (year)** | **Size (% Male)** | **Age, y**  **mean (SD)** | **Mortality follow-up (yrs)** | **Continuous or categorical** | **Statistic reported** | **No. deaths** | **Results** | **AUC≥0.7 (Max 1)** | **Study quality (SIGN checklist)** |
| --- | --- | --- | --- | --- | --- | --- | --- | --- | --- |
| Chao (2018) | 7713 (n/s) | n/s (aged ≥65) | 7 | Continuous | AUC^‡1^ | 4140 | 0.731 (0.724-0.739) | Yes | + |
| Romero-Ortuno and Soraghan (2014) | 7058 (43.3%) | M: 80.4 (4.6) F: 81.1 (4.9) | 2 | Continuous | AUC^?1^ | M: 239  F: 132 | M: 0.70 (0.66-0.74) F: 0.65 (0.61-0.7) | Yes No | ++ |
| Ding (2017) | 4638 (44.6%) | 74.0 (6.3) | 2 | Continuous | c-statistic^*1^ | 278 | 0.750 (0.721-0.779)^b^ | Yes | 0 |
| Li (2015) | 3985 (0%) | 69.4 (8.9) | 3 | Continuous^a^ | AUC^‡1^ | 107 | 0.80 (0.76-0.85) | Yes | ++ |
| Zucchelli (2019) | 3363 (35.1%) | 74.7 (11.2) | 3 | Continuous | AUC^*3^ | 477 | 0.84 (0.82-0.86) | Yes | + |
| Widagdo (2015) | 2087 (n/s) | n/s (aged ≥65) | 3 | Continuous | AUC^*1^ | 346 | 0.66 (0.63-0.69)^b^ | No | 0 |
| Thompson (2019) | 909 (45%) | 74.4 (6.2) | 4 | Continuous | AUC^‡1^ | 86 | 0.73 (0.67‐0.79) | Yes | ++ |
| Chao (2018) | 7713 (n/s) | n/s (aged ≥65) | 7 | Categorical | AUC^‡1^ | 4140 | 0.721 (0.713-0.728) | Yes | + |
| Woo (2012) | 4000 (50%) | n/s (aged ≥65) | 4 | Categorical | AUC^*1^ | M: 350  F: 137 | M: 0.632 (0.600-0.664) F: 0.612 (0.564-0.660) | No No | + |
| Li (2015) | 3985 (0%) | 69.4 (8.9) | 3 | Categorical | AUC^‡1^ | 107 | 0.80 (0.75-0.84) | Yes | + |
| Widagdo (2015) | 2087 (n/s) | n/s (aged ≥65) | 3 | Categorical | AUC^*1^ | 346 | 0.60 (0.57-0.63)^b^ | No | 0 |
| Thompson (2019) | 909 (45%) | 74.4 (6.2) | 4 | Categorical | AUC^‡1^ | 86 | 0.73 (0.68‐0.79) | Yes | ++ |

^a^Used quintiles rather than items

^b^No confidence interval provided so estimated standard error using formula (3) page 2771 from: Debray et al. A framework for meta-analysis of prediction model studies with binary and time-to-event outcomes. Statistical Methods in Medical Research 2019:28(9)2768-2786 <https://doi.org/10.1177/0962280218785504>

Level of adjustments used to compute AUC statistic were: ^†^: age and sex; ^‡^: age and/or sex plus other confounders; ^*^: none specified; ^?^: undetermined

Model used to generate ROC were: ^1^: logistic regression; ^2^: Cox regression; ^3^: other; ^4^: not specified

*AUC can be approximated using sensitivity and specificity: (sensitivity + specificity)/2

## Appendix Table 7. Characteristics of individual studies with data on FP

| **Author (year)** | **Size (% Male)** | **Age, y**  **mean (SD)** | **Mortality follow-up (yrs)** | **Continuous or categorical** | **Statistic reported** | **No. deaths** | **Results** | **AUC≥0.7 (Max 1)** | **Study quality (SIGN checklist)** |
| --- | --- | --- | --- | --- | --- | --- | --- | --- | --- |
| Romero-Ortuno and Soraghan (2014) | 7058 (43.3%) | M: 80.4 (4.6) F: 81.1 (4.9) | 2 | Continuous | AUC^?1^ | M: 239  F: 132 | M: 0.69 (0.65-0.73) F: 0.65 (0.61-0.7) | No No | ++ |
| Ding (2017) | 4638 (44.6%) | 74.0 (6.3) | 2 | Continuous^a^ | c-statistic^*1^ | 278 | 0.750 (0.721-0.779)^b^ | Yes | 0 |
| Li (2015) | 3985 (0%) | 69.4 (8.9) | 3 | Continuous | AUC^‡1^ | 107 | 0.79 (0.74-0.84) | Yes | ++ |
| Zucchelli (2019) | 3363 (35.1%) | 74.7 (11.2) | 3 | Continuous | AUC^*3^ | 477 | 0.80 (0.78-0.82) | Yes | + |
| Widagdo (2015) | 2087 (n/s) | n/s (aged ≥65) | 3 | Continuous | AUC^*1^ | 205 | 0.63 (0.59-0.67)^b^ | No | 0 |
| Chao (2018) | 1642 (n/s) | n/s (aged ≥65) | 7 | Continuous | AUC^‡1^ | 754 | 0.766 (0.749-0.782) | Yes | + |
| Thompson (2019) | 909 (45%) | 74.4 (6.2) | 4 | Continuous | AUC^‡1^ | 86 | 0.73 (0.68‐0.79) | Yes | ++ |
| Woo (2012) | 4000 (50%) | n/s (aged ≥65) | 4 | Categorical | AUC^*1^ | M: 350  F: 137 | M:0.641 (0.608-0.673) F:0.602 (0.550-0.653) | No No | + |
| Li (2015) | 3985 (0%) | 69.4 (8.9) | 3 | Categorical | AUC^‡1^ | 107 | 0.79 (0.74-0.83) | Yes | + |
| Widagdo (2015) | 2087 (n/s) | n/s (aged ≥65) | 3 | Categorical | AUC^*1^ | 205 | 0.57 (0.53-0.61)^b^ | No | 0 |
| Chao (2018) | 1642 (n/s) | n/s (aged ≥65) | 7 | Categorical | AUC^‡1^ | 754 | 0.754 (0.736-0.771) | Yes | + |
| Thompson (2019) | 909 (45%) | 74.4 (6.2) | 4 | Categorical | AUC^‡1^ | 86 | 0.71 (0.66‐0.78) | Yes | ++ |

^a^Used factor score from CFA

^b^No confidence interval provided so estimated standard error using formula (3) page 2771 from: Debray et al. A framework for meta-analysis of prediction model studies with binary and time-to-event outcomes. Statistical Methods in Medical Research 2019:28(9)2768-2786 <https://doi.org/10.1177/0962280218785504>

Level of adjustment: ^†^: age and sex; ^‡^: age and/or sex plus other confounders; ^*^: none specified; ^?^: undetermined

Model used to generate ROC were: ^1^: logistic regression; ^2^: Cox regression; ^3^: not specified

*AUC can be approximated using sensitivity and specificity: (sensitivity + specificity)/2

## Appendix Table 8. Rationale given by authors for continuous and categorical labels

| **Author (year)** | **Continuous and/or categorical** | **Rationale** |
| --- | --- | --- |
| Chao (2018) | Continuous and categorical | p9 - "The predictive power of three frailty indices in continuous scales or dichotomous statues regarding mortality..." |
| Li (2015) | Continuous and categorical | p1 - "(1) investigated the relationship with adverse health outcomes by increasing per one-fifth (i.e., 20%) of the FI and PF; (2) trichotomized the FI based on the overlap in the density distribution of the FI by the three groups (robust, pre-frail and frail) which were defined by the PF" |
| Widagdo (2015) | Continuous and categorical | In ‘Statistical analysis’ – “This analysis used a dichotomised frailty status (frail and non-frail); for those measures that had a pre-frail group, this was combined into non-frail… The AUC values for the full model (i.e. multilevel or continuous scale) of the frailty measures were also assessed.” |
| Thompson (2019) | Continuous and categorical | In Methods – “Three iterations of the FP were used: a Continuous FP; … and a 3‐Category FP… Three iterations of the FI were used: a Continuous FI; … and a 3‐Category FI” |
| Romero-Ortuno and Soraghan (2014) | Continuous | p14 – Figures showing ROC curve suggest continuous scores were used |
| Ding (2017) | Continuous | p4 - "1 unit of increase in…" |
| Zucchelli (2019) | Continuous | p3 - "The AUC was obtained using non-parametric ROC analysis, including the different indicators as continuous variables" |
| Kusumastuti (2017) | Categorical | p3 - "health indicators were dichotomized into…, FI score of <0.2 and >0.3, Frailty Phenotype score of 0 and >1" |
| Woo (2012) | Categorical | p7 - Figures showing ROC curve suggest categorical scores were used |

## Appendix Table 9. Details of the frailty phenotype (FP)

| **Author, year** | **Exhaustion (poor endurance and energy)** | **Physical activity** | **Weight loss/shrinking** | **Weakness** | **Gait speed/slowness** | **Cutoff** |
| --- | --- | --- | --- | --- | --- | --- |
| **Fried et al., 2001** | **Self‐rated exhaustion 2 items from the CES-D Scale [35]: “I feel that everything I do is an effort” and “I cannot get going”  Feel that everything was an effort or could not get going for ≥3 days in the last week** | **Low physical activity  Low energy expenditure (male < 383 kcal/week, female < 270 kcal/week)** | **Unintentional weight loss ≥4.5 kg or ≥5% within one year (self‐report)** | **Low grip strength  In the lowest 20% at baseline (adjusted for sex and BMI)** | **Walking time/15 ft (4.5 m)  Slowest 20% at baseline (adjusted for sex and height)** | **Frail, ≥3 variables present; Pre‐frail, 1–2 variables present; Non‐frail, no variables present.** |
| Li et al., 2015 | The Medical Outcomes Study 36-item Short Form Survey (SF-36) vitality component's four questions on whether they felt worn out, felt full of life, felt tired, or had a lot of energy. For responses to feeling worn out and feeling tired, the scores were 100 points for none of the time, 75 points for a little of the time, 25 points for most of the time, and 0 point for all of the time. The scores were reversely coded for responses to having a lot of energy and feeling full of life. The lowest quartile of poor endurance and exhaustion obtained 1 point. | Lowest 25% in the number of days that in the past 30 days they had walked at least 20 minutes. | Unintentional loss of >10lb in the past year | The Medical Outcomes Study 36-item Short Form Survey (SF-36) physical functioning component to assess the limitations in 10 activities such as running, bathing, bending, and walking. In each of the activities, participants received 0 point if they reported they were limited a lot, and 50 points if they were limited a little, and 100 points if they were not limited at all respectively. The lowest quarter of slowness and weakness received 2 points | | Frail, ≥3 variables present; Pre‐frail, 1–2 variables present; Non‐frail, no variables present. |
| Widagdo et al., 2015 | Self‐rated exhaustion (CES‐D scale)  Feel that everything was an effort or could not get going for ≥3 days in the last week | Walking for exercise or recreation in the past 2 weeks | BMI < 20 kg/m2 | Grip strength in the lowest 20% at baseline (adjusted for sex and BMI) | Slowest 20% at baseline (adjusted for sex and height) using TUG test (8ft/2.4m) | Frail, ≥3 variables present; Pre‐frail, 1–2 variables present; Non‐frail, no variables present. |
| Ding et al., 2017 | Binary variable based on a positive reply to either or both of 2 items in CES-D scale on whether the respondent "felt everything they did during the past week was an effort" and " could not get going much of the time in the past week" | Lowest 4 categories of physical activity (sedantary) | Weight loss of >5kg from waves 0 to 2(on average 4 years) | Dominant hand grip strength in kg which is multiplied by 1.5 for women (1.5 represents the relative difference in gender-specific and population-dependent values for grip strength). After that the values are reversed through multiplying by -1. | The average gait speed (m/s) of 2 attempts at walking distance of 2.4m multiplied by -1 | n/s |
| Zucchelli et al., 2019 | Participants were asked if they experienced fatigue in the last three months. (Positive answer) | Participants were asked “do you regularly engage in light exercise (e.g. walking along roads or in parks, walking in the woods, short bicycle rides, light aerobics, golf)?” and “do you regularly engage in moderate to intense exercise, now or previously (e.g. jogging, long power walks, heavy-duty gardening, long bicycle rides, high-intensity aerobics, long distance ice skating, swimming, ball sports (not golf) or other similar activity)?” (“less than 2-3 times/month” for both questions) | Participants were asked “did you suffer from any weight loss in the last 3 months?”. (Reporting of loss of at least 1 kilogram) | Grip strength in the lowest 20th percentile, adjusted for sex and body-mass-index. Participants were asked to employ maximum force while squeezing the handle of an electronic dynamometer (Grippit ®), one time for each hand. The best value was recorded. | Slowest 20th percentile, adjusted for sex and height. Participants were asked to walk at usual speed over a 6-meter straight path. If the subjects reported slow walking speed or if, because of space issues, the full-length track was not available, a 2.4-meter was used. Walking speed was registered as m/s. A value of 0 was accounted if the subject was unable to walk. | Frail, ≥3 variables present; Pre‐frail, 1–2 variables present; Non‐frail, no variables present. |
| Woo et al., 2012 | Self-reported no energy | Lowest 25% for Physical Activity Scale for the Elderly (PASE) score | BMI < 18.5 kg/m2 | Grip strength in the lowest 25%, measured using a dynamometer (JAMAR hand dynamometer 5030JI; Sam- mons Preston, Bolingbrook, IL). The average of two readings on the right and left side was used. | Slowest 25% using TUG test (6m) | Frail, ≥3 variables present; Pre‐frail, 1–2 variables present; Non‐frail, no variables present. |
| Chao et al., 2018 | Yes to either of two CES-D items: (i) Felt that everything I did was an effort in last week. (ii) Could not get going in last week. | Lowest 20% (sex-specific) in frequency of three intensities of activity | BMI <18.5 kg/m2 | Grip strength in the lowest 20% (adjusted for sex and BMI) | Time to walk eight feet, converted to time to walk 15 feet categorized by height and sex | Dichotomised as Frail (≥3) or not. |
| Romero-Ortuno and Soraghan, 2014 | “In the last month, have you had too little energy to do things you wanted to do? (yes/no).” | “How often do you engage in activities that require a low or moderate level of energy such as gardening, cleaning the car, or going for a walk?.” (“one to three times a month” or “hardly ever or never”) | “What has your appetite been like” ("diminuation in desire for food") or “So have you been eating more or less than usual?” ("less") | Highest of four consecutive dynamometer measurements of handgrip strength (two from each hand), applying gender and body mass index cutoffs set by Fried and associates | “Because of a health problem, do you have difficulty [expected to last more than 3 months] walking 100 meters” or “… climbing one flight of stairs without resting” | Frail, ≥3 variables present; Pre‐frail, 1–2 variables present; Non‐frail, no variables present. |
| Thompson, 2019 | Original method | Australian Bureau of Statistics National Health Survey (<100 METs per week) | Weight Loss: > 10% weight loss over four years (clinic measurement) | Original method | Self-report ‘a lot’ to health limits walking 100m ( 36-Item Short Form Health Survey Q11) | Frail, ≥3 variables present; Pre‐frail, 1–2 variables present; Non‐frail, no variables present. |

## Appendix Table 10. Details of the frailty index (FI)

| **Author, year** | **Item no.** | **Included items** | | | **Cutoff** |
| --- | --- | --- | --- | --- | --- |
| Li et al., 2015 | 34 | 1. Taking/taken five or more medications 2. Has chronic bronchitis or emphysema 3. Has osteoarthritis or degenerative joint disease 4. Has rheumatoid arthritis 5. Suffers from stroke 6. Has ulcerative colitis or Crohn's disease 7. Has celiac disease 8. Has Parkinson's disease 9. Has multiple sclerosis 10. Has cancer 11. Has diabetes (type-1) | 12. Has hypertension 13. Has heart disease 14. Has high cholesterol 15. Self rating of health 16. Limitations in vigorous activities 17. Limitations in moderate activities 18. Limitations in lifting or carrying in groceries 19. Limitations in climbing one flight of stairs 20. Limitations bending, kneeling or stooping 21. Limitations walking one hundred yards 22. Limitations in bathing or dressing yourself | 23. Needs arms to help stand up from a chair 24. Number of days to walk at least 20 minutes in the past 30 days 25. Self rating of mobility 26. Self rating of self-care 27. Self rating of usual activities 28. Feels full of life 29. Has a lot of energy 30. Feels worn out 31. Feels tired 32. Self rating of pain/discomfort 33. Unintentional weight loss of 10 pounds 34. Times of visiting a healthcare provider to get medical care in the past year | Frail (>0.35),  Pre-frail (0.20–0.35), Non-frail (<0.20) |
| Widagdo et al., 2015 | 39 | 1. Live alone  2. Self‐rated health (Poor = 1; Fair = 0.75; Good = 0.5; Very good = 0.25; Excellent = 0)  3. Arthritis  4. Asthma  5. History of heart attack  6. Hypertension  7. Migraine  8. Parkinson's disease  9. History of stroke  10. Thyroid disease  11. Ear, nose or throat problem  12. Mental disorder  13. Genito‐urinary problem | 14. Diabetes  15. Cancer  16. Chest pain  17. Constipation  18. Dental problem  19. Sleep problem (Rarely/never = 0; Sometimes = 0.5; Often/almost always = 1)  20. Spinal problem  21. Hearing difficulty  22. Eye trouble  23. Skin problem  24. Problem with hands shaking (No difficulty at all or a little difficulty = 0; Some/a lot of difficulty = 1) 25. Stooping/crouching/kneeling problem (No difficulty at all or a little difficulty = 0; Some/a lot of difficulty or just unable to do = 1) | 26. Difficulty with bathing  27. Difficulty with personal grooming  28. Difficulty with dressing  29. Difficulty with eating  30. Difficulty with toileting  31. Difficulty with going out  32. Difficulty with moving around  33. Difficulty with laundry/linen  34. Difficulty with housework  35. Difficulty with preparing meals  36. Difficulty with using telephone  37. Difficulty with managing money  38. Difficulty with using public transport  39. Difficulty with shopping | Frail (>0.25) |
| Ding et al., 2017^1^ | 30(29?) | 1. Chronic illness: Hypertension 2. Chronic illness: Myocardial Infarction 3. Chronic illness: Congestive heart failure 4. Chronic illness: Diabetes Mellitus 5. Chronic illness: Stroke 6. Chronic illness: Arthritis 7. Chronic illness: Cancer 8. Chronic illness: Obesity 9. Psychological condition: Dementia | 10. Psychological condition: Feeling depressed (CESD) 11. Psychological condition: Feeling effortful (CESD) 12. Psychological condition: Feeling happy (CESD): reverse 13. Psychological condition: Feeling lonely (CESD) 14. Psychological condition: Could not get going (CESD) 15. Poor self-rated health 16. Mobility: walking 100 yards 17. Mobility: getting up from chair 18. Mobility: climbing stairs 19. Mobility: lifting weights | 20. BADL: dressing 21. BADL: walking 22. BADL: bathing 23. BADL: eating 24. BADL: toileting 25. IADL: shopping 26. IADL: taking medication 27. IADL: doing housework 28. IADL: managing finances 29. Weak grip strength | Frail (>0.25),  Pre-frail (0.08 - 0.25), Non-frail (<0.08) |
| Zucchelli et al., 2019 | 45 | 1. Hypertension 2. Thyroid disorders 3. Anaemia 4. Atrial Fibrillation 5. Blindness and other visual impairments 6. Cerebrovascular diseases 7. Chronic kidney disease 8. Chronic Obstructive Pulmonary Disease, emphysema and chronic bronchitis 9. Deafness and other hearing impairments 10. Dementia 11. Depression and other mood disorders 12. Diabetes 13. Heart failure 14. Inflammatory bowel diseases 15. Ischemic heart disease | 16. Osteoporosis 17. Parkinson disease and parkinsonisms 18. Peripheral arterial diseases 19. Peripheral neuropathies 20. Sleep disorders 21. Solid neoplasm 22. Inability to autonomously take and prepare medications 23. Inability to autonomously shop 24. Inability to autonomously cook and prepare meals 25. Inability to autonomously do house chores  26. Inability to autonomously do the laundry 27. Inability to autonomously manage finances 28. Inability to autonomously use the telephone 29. Inability to autonomously use means of transportation 30. Minimental State Examination score 1 if < 18, 0.5 if 18 ≤ score < 23, 0.25 if ≤24 score < 27, 0 if ≥ 27 | 31. Inability to autonomously bath 32. Inability to autonomously dress 33. Inability to autonomously use the toilet 34. Incontinence 35. Inability to autonomously move in and out from bed or chair 36. Inability to autonomously feed him-/herself 37. Usage of stick during walking 38. Reporting dyspnoea 39. Evidence of swollen legs at physical examination 40. Reporting anxiety symptoms 41. Self-reporting appetite loss 42. Self-reporting sense of worthlessness 43. Reporting pain 44. Feeling older than actual age 45. Self-reporting low quality of life | Frail (>0.25),  Pre-frail (0.08 - 0.25), Non-frail (<0.08) |
| Woo et al., 2012 | 47 | Self-reported health, history of falls in the past 12 months, history of osteoporotic fractures, presence of back pain limiting activities, clumsiness in walking, clumsiness using hands, number of prescription medica- tions, and any difficulty performing activities of daily liv- ing (walking 2–3 blocks outside on level ground, climbing 10 steps without resting, preparing own meals, doing heavy housework such as scrubbing floors or washing windows, and doing own shopping for groceries or clothes). The presence or absence of disease was based on participant report of diagnosis by their doctor. Depressive symptoms were assessed using the Geriatric Depression Scale (GDS),21 which has been validated in elderly Chinese adults, with a score of 8 or greater representing depressive symptoms. Cognitive impairment was assessed using the Cognitive Screening Instrument for Dementia (CSID) with a cutoff of 28.4. Physical activity level was assessed the Physical Activity Scale for the Elderly (PASE).  Height, weight, time and number of steps taken to walk 6 m, grip strength, blood pressure, and ankle brachial index (ABI) were measured on the same day that the ques- tionnaire was administered. Step length was calculated by dividing 6 m by the total number of steps taken. Body weight was measured with participants wearing a light gown using the Physician Balance Beam Scale (Healthometer, Alsip, IL). Height was measured using the Holtain Har- penden stadiometer (Holtain Ltd, Crosswell, UK). BMI was calculated by dividing weight in kg by the square of height in meters. Grip strength was measured using a dynamometer (JAMAR hand dynamometer 5030JI; Sam- mons Preston, Bolingbrook, IL). The average of two read- ings on the right and left side was used. Blood pressure was measured twice in the supine portion using a mercury sphygmomanometer and the average used. Duplicate mea- sures of supine blood pressure in the right arm and both ankles were taken using a standard mercury sphygmoma- nometer and an 8-MHz Doppler probe (Pocket Doppler Model 841-A; Parks Medical Electronics, Inc., Aloha, OR). ABI was calculated for each leg by dividing the pos- terior tibial systolic pressure in each lower extremity by the upper extremity pressure. The current standard for diagnosing peripheral vascular disease is defined as an ABI less than 0.90. An ABI of less than 0.90 is 95% sensitive and 99% specific for angiographically diagnosed peripheral arterial diseease. The lower ABI of the 2 was used to determine the extent of ischaemic disease. | | | Cutoff points used 0.05, 0.1, 0.15, 0.2, 0.25 |
| Chao et al., 2018^2^ | 24 | 1. Problems getting dressed 2. Problems with bathing 3. Urinary incontinence 4. Toileting problems 5. Impaired mobility 6. Falls 7. Feeling sad, blue, depressed  8. Tiredness all the time | 9. Depression (clinical impression) 10. Changes in general mental functioning 11. Memory changes 12. Sleep changes 13. History relevant to cognitive impairment or loss 14. Arterial hypertension 15. Seizures, generalized 16. Headache | 17. Cerebrovascular problems 18. History of diabetes mellitus 19. Musculoskeletal problems 20. Congestive heart failure 21. Lung problems or respiratory problems 22. Malignant disease 23. Other medical history 24. Proxy memory rating | Frail (>0.2) |
| Romero-Ortuno and Soraghan, 2014 | 70 | 1. Self‐Rated Health  2. Hospitalization in Past Year 3. Heart attack  4. Stroke or CVD  5. High blood cholesterol  6. Diabetes mellitus or high blood sugar 7. Chronic lung disease  8. Asthma  9. Long‐term illness  10. Arthritis 11. Osteoporosis  12. Cancer  13. High blood pressure  14. Stomach or duodenal ulcer 15. Parkinson disease  16. Cataracts  17. Heart trouble or angina  18. Hip or femoral fracture 19. Falling down  20. Sleeping problems  21. Dizziness  22. Swollen legs 23. Stomach or intestine problems | 24. Incontinence  25. Persistent cough  26. Require dentures 27. Difficulty biting on hard foods  28. Problems with eyesight  29. Hearing problems  30. Pain in any joint 31. Breathlessness  32. Climbing several flights of stairs  33. Stooping/kneeling/crouching  34. Sitting for about 2 hours  35. Reaching or extending arms 36. Pulling/pushing large objects  37. Lifting/carrying weights >5 kg  38. Walking 100 m  39. Picking up a small coin from table 40. Dressing  41. Walking across a room  42. Getting up from a chair  43. Bathing or showering 44. Eating  45. Getting in or out of bed  46. Preparing a hot meal | 47. Using the toilet 48. Using a map to get around  49. Making telephone calls  50. Taking medications  51. Shopping for groceries 52. Managing money  53. Vigorous activities  54. Moderate activities  55. Doing work around house/garden 56. Limitations with activities  57. Orientation  58. Mathematical performance  59. Delayed recall test  60. Verbal fluency score 61. Suicidality  62. Trouble sleeping  63. Depression  64. Interest 65. Appetite  66. Fatigue 67. Pessimism  68. Concentration 69. Lack of enjoyment  70. Fear of falling down | n/s |
| Thompson. 2019 | 34 | 1. Angina  2. Heart attack  3. Osteoporosis  4. Osteoarthritis  5. Rheumatoid and any other arthritis  6. Stroke or TIA  7. Diabetes  8. Any mental health problem  9. Systolic blood pressure  10. Diastolic blood pressure  11. 10% weight loss over 4 years | 12. FEV1/FVC post ratio  13. Weak grip strength  14. Falls  15. Hospital emergency admission  16. Low activity level (<100 METs per week)  17. Healthy as anybody I know  18. Health is excellent  19. Self-reported health  20. Health limits lifting or carrying groceries  21. Health limits climbing several flights of stairs  22. Health limits climbing one flight of stairs  23. Health limits bending, kneeling or stooping | 24. Health limits walking more than 1km  25. Health limits walking 100m  26. Felt lonely  27. Felt that could not get going  28. Difficulty keeping mind on what you were doing  29. Felt everything was an effort  30. Physical & emotional problems interfered with social activities  31. Felt full of life  32. Felt calm and peaceful  33. Felt worn out  34. Felt tired | Frail ( >0.21),  Pre-frail (0.10 - 0.21),  Non-frail (<0.10) |

^1^Rationale for no. of items <30: Manuscript says 30 items, but the FI list suggests 29 items. (List obtained from a previous publication by the same author using the same dataset - Ding et al. Multidimensional predictors of physical frailty in older people: identifying how and for whom they exert their effects. Biogerontology. 2017; 18(2):237-252)

^2^Rationale for no. of items <30: The authors could only not retrieve the same variables to approximate the 70-items from the original study they tried to recreate.

## Appendix Table 11. Domains included in the FI

| **Author (year)** | **Item no.** | **Energy** | **Physical activity** | **Weight loss/BMI** | **Strength**^a^ | **Gait**^b^ | **Cognition** | **Mood** | **ADL** | **Self-rated health** | **Hearing and vision** | **Incontinence** | **Medication** | **Sleep** | **Hospitalisation** | **Comorbidities** | **Symptoms** | **Social support** | **Falls** |
| --- | --- | --- | --- | --- | --- | --- | --- | --- | --- | --- | --- | --- | --- | --- | --- | --- | --- | --- | --- |
| Chao (2018) | 24 | X |  |  |  |  | X | X | X |  |  | X |  | X |  | X | X |  | X |
| Romero-Ortuno and Soraghan (2014) | 70 | X | X |  | X | X | X | X | X | X | X | X | X | X | X | X | X |  | X |
| Ding (2017) | 30 (29?) | X |  |  | X | X | X | X | X | X |  |  | X |  |  | X |  |  |  |
| Woo (2012) | 47 |  | X | X | X | X | X | X | X | X |  |  | X |  |  | X | X |  | X |
| Li (2015) | 34 | X | X | X | X | X |  | X | X | X |  |  | X |  | X | X | X |  |  |
| Zucchelli (2019) | 45 |  |  |  |  | X | X | X | X | X | X | X | X | X |  | X | X |  |  |
| Widagdo (2015) | 39 |  |  |  | X | X | X |  | X | X | X | X |  | X |  | X | X |  |  |
| Thompson (2019) | 34 | X | X | X | X | X | X | X | X | X |  |  |  |  | X | X | X |  | X |

^a^Items like grip strength and difficulty lifting weights over 10 lbs

^b^Items like gait speed, can you walk 100m, difficulty with moving around, and usage of walking stick

List of domains adapted from: Theou O, Brothers TD, Peña FG et al. Identifying Common Characteristics of Frailty Across Seven Scales. J Am Geriatr Soc 2014;62:901–6.

## Appendix Figure 1. Plot of discriminative ability as assessed by Area Under the Curve (AUC) against number of events by methodological quality


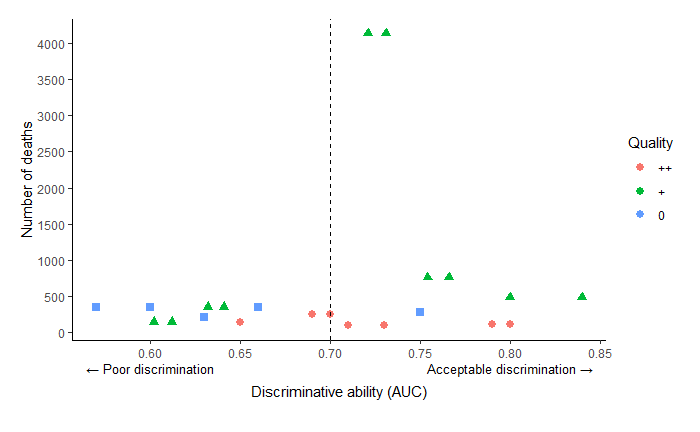


## Appendix Figure 2. Discriminative ability as assessed by Area Under the Curve (AUC) for Frailty Index (FI) continuous instruments arranged by total number of domains


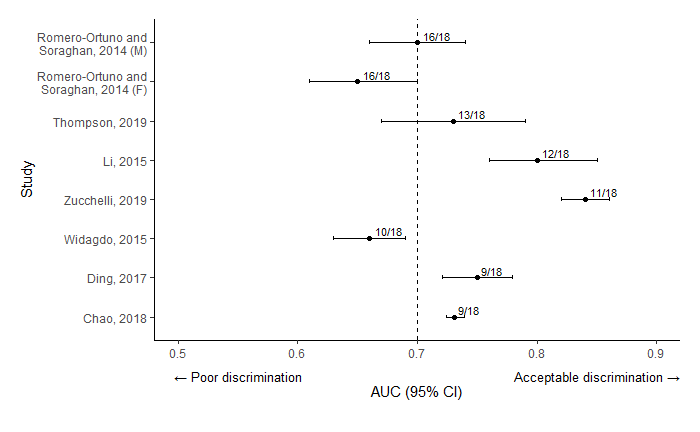


## Appendix Figure 3. Discriminative ability as assessed by Area Under the Curve (AUC) for Frailty Index (FI) categorical instruments


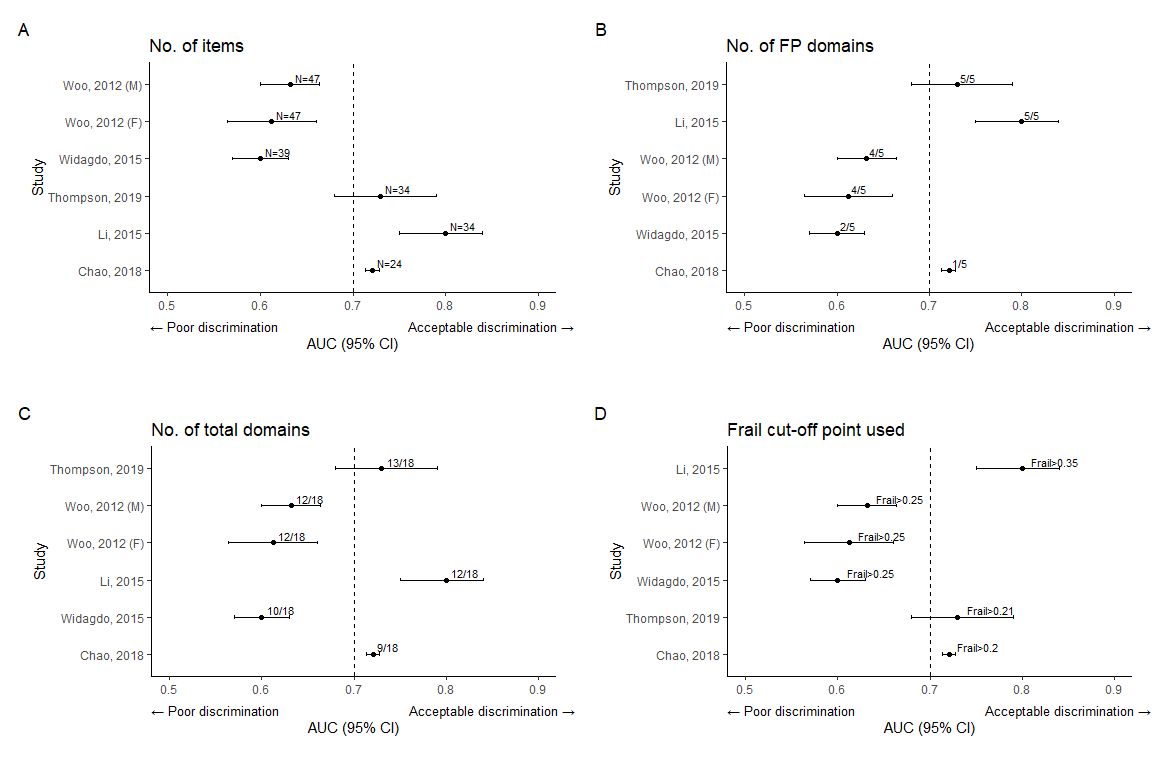


Arranged by: no. items (A); no. FP domains (B); no. total domains (C); and frail cut-off point used (D)
